# Electrophysiological effects of nicotinic and electrical stimulation of intrinsic cardiac ganglia in the absence of extrinsic autonomic nerves in the rabbit heart

**DOI:** 10.1016/j.hrthm.2018.05.018

**Published:** 2018-11

**Authors:** Emily Allen, John H. Coote, Blair D. Grubb, Trevor F.C. Batten, Dainius H. Pauza, G. André Ng, Kieran E. Brack

**Affiliations:** ∗ Department of Cardiovascular Sciences, University of Leicester, Glenfield Hospital, Leicester, United Kingdom; † NIHR Leicester BRC, Glenfield Hospital, Leicester, United Kingdom; ‡ Institute of Life and Human Sciences, University of Liverpool, Liverpool, United Kingdom; § AF3, Leeds Trinity University, Leeds, United Kingdom; ‖ Institute of Anatomy, Lithuanian University of Health Sciences, Kaunas, Lithuania

**Keywords:** Choline acetyltransferase, Electrophysiology, Ganglionic plexus, Heart, Intrinsic cardiac ganglia, Neuronal nitric oxide synthase, Tyrosine hydroxylase

## Abstract

**Background:**

The intrinsic cardiac nervous system is a rich network of cardiac nerves that converge to form distinct ganglia and extend across the heart and is capable of influencing cardiac function.

**Objective:**

The goals of this study were to provide a complete picture of the neurotransmitter/neuromodulator profile of the rabbit intrinsic cardiac nervous system and to determine the influence of spatially divergent ganglia on cardiac electrophysiology.

**Methods:**

Nicotinic or electrical stimulation was applied at discrete sites of the intrinsic cardiac nerve plexus in the Langendorff-perfused rabbit heart. Functional effects on sinus rate and atrioventricular conduction were measured. Immunohistochemistry for choline acetyltransferase (ChAT), tyrosine hydroxylase, and/or neuronal nitric oxide synthase (nNOS) was performed using whole mount preparations.

**Results:**

Stimulation within all ganglia produced either bradycardia, tachycardia, or a biphasic brady-tachycardia. Electrical stimulation of the right atrial and right neuronal cluster regions produced the largest chronotropic responses. Significant prolongation of atrioventricular conduction was predominant at the pulmonary vein-caudal vein region. Neurons immunoreactive (IR) only for ChAT, tyrosine hydroxylase, or nNOS were consistently located within the limits of the hilum and at the roots of the right cranial and right pulmonary veins. ChAT-IR neurons were most abundant (1946 ± 668 neurons). Neurons IR only for nNOS were distributed within ganglia.

**Conclusion:**

Stimulation of intrinsic ganglia, shown to be of phenotypic complexity but predominantly of cholinergic nature, indicates that clusters of neurons are capable of independent selective effects on cardiac electrophysiology, therefore providing a potential therapeutic target for the prevention and treatment of cardiac disease.

## Introduction

In recent decades, a rich network of intrinsic cardiac nerves that converge to form distinct ganglia and extend across the heart has been documented in mammalian species including dog, cat, pig, guinea pig, mouse, as well as human.^1^ Evidence suggests that activity within these ganglia may result in cardiac changes both locally and in other regions of the myocardium, independent of extrinsic autonomic nerves.^1^, ^2^, ^3^, ^4^ This has given rise to the notion that such an intrinsic cardiac nervous system (ICNS) acts as the heart’s “*little brain*,” capable of influencing cardiac function^5^, ^6^ even in the absence of extrinsic autonomic input, although this has not been directly tested.

The somata of intrinsic neurons occur within the epicardium and spread widely over the walls of all chambers of the heart, forming an interconnected “ganglionic plexus.”^7^, ^8^, ^9^ Historically, it was presumed that intrinsic cardiac ganglia were merely parasympathetic neuronal relay stations^1^, ^10^; however, a recent review by Wake and Brack^1^ showed evidence that these ganglia are neurophenotypically and neurochemically diverse.^9^, ^11^, ^12^ This would suggest that ganglia are also functionally diverse and could provide key knowledge to understand the remodeling of ganglia in cardiac diseases such as heart failure and atrial/ventricular arrhythmias.^13^, ^14^

Our understanding of the cardiac regulatory functions of this dispersed epicardial ganglionic plexus is limited, presently depending either on electrophysiological studies of cells in isolated atria or on in situ heart studies in anesthetized dogs^6^ with intact extrinsic autonomic nerves. Therefore, we considered that the Langendorff-perfused rabbit heart^15^ could overcome these limitations and provide a valuable model to study the integrative action of the ICNS and its significance on cardiac performance in the isolation of circulating and extraneuronal factors.

Therefore, the primary objective of the present study was to determine the influence of spatially divergent ganglia on cardiac electrophysiology in the absence of extrinsic autonomic nerve influence. We tested the effects of nicotinic and electrical stimulation, which have been the main methods used in previous studies^3^, ^16^, ^17^, ^18^; and unlike earlier studies, we compared the effects of applying stimuli to loci within 4 regions while measuring effects on cardiac electrophysiology. In addition, with the aim of providing a complete picture of the neurotransmitter/neuromodulator profile of the rabbit intrinsic cardiac network, immunofluorescent labeling was performed using whole mount atrial preparations.

## Methods

### Ethical statement

All procedures were performed using adult male New Zealand White rabbits (n = 46; weighing 1.5–3.4 kg) in accordance with the UK Animals (Scientific Procedures) Act 1985, the *Guide for the Care of Use of Laboratory Animals* published by the US National Institutes of Health (NIH Publication No. 85-23, revised 1985), and the European Union Directive on the protection of animals for scientific research (2010/63/EU). Local ethics approval was obtained from the Animal Welfare and Ethical Review Body of the University of Leicester under the Home Office Project Licence PPL 70/8501.

### Animal preparation

Of the 46 animals used in this study, 28 were used to study the influence of spatially divergent ganglia on cardiac electrophysiology and a separate group of 18 was used for immunohistochemical analysis. All animals were premedicated, and after stable sedation, animals were killed (see the Supplement).

### Isolation of the noninnervated heart preparation

Non-innervated hearts were isolated as previously described.^19^, ^20^ In brief, animals were premedicated and killed. Hearts were rapidly excised, placed into ice cold Tyrode’s solution, and retrogradely perfused through the ascending aorta in conditions of constant flow Langendorff mode (40 mL/min) (see the Supplement).

### Nicotinic stimulation of intrinsic cardiac ganglia

**Figure 1:**
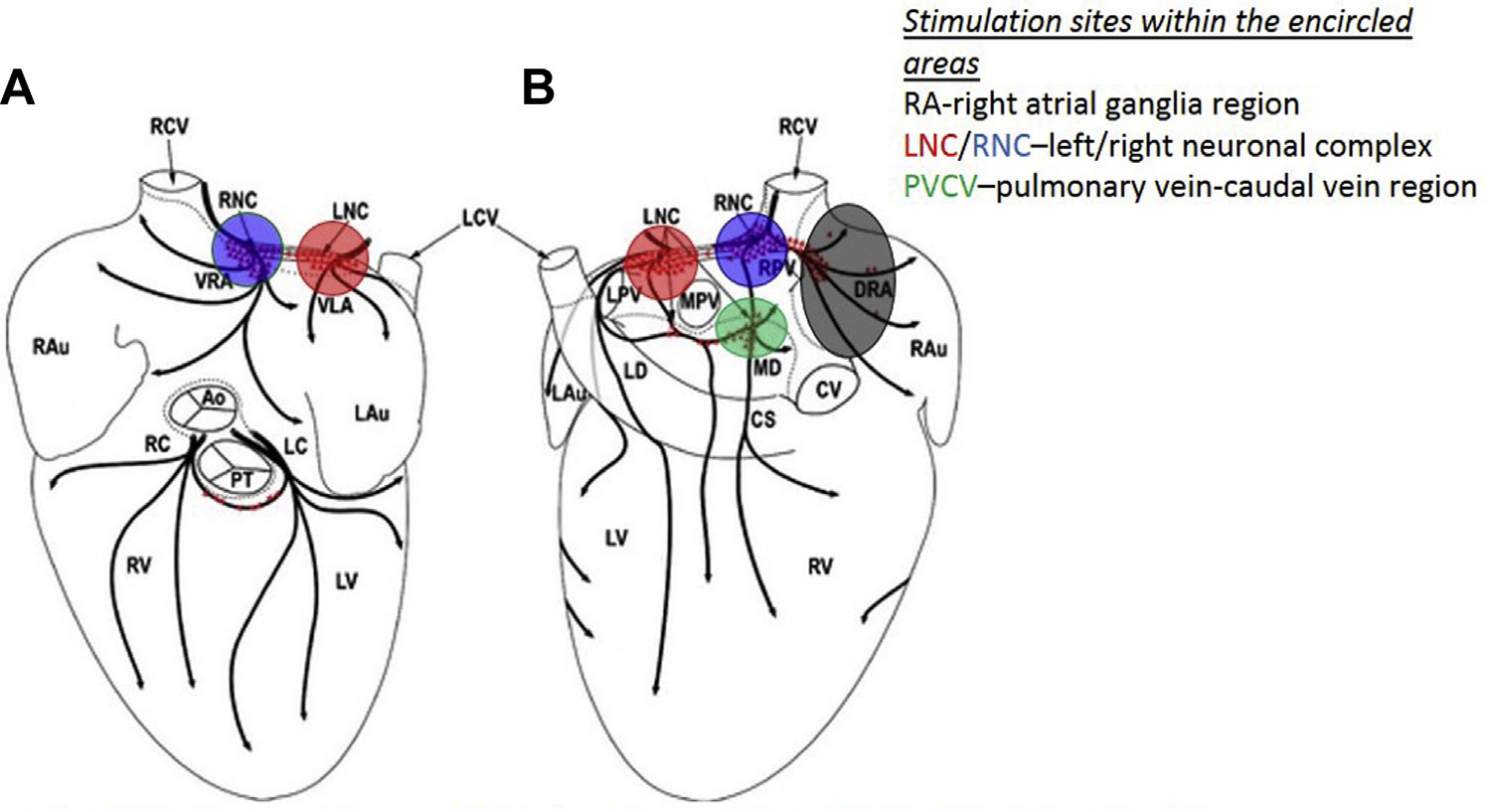
Anterior (**A**) and posterior (**B**) views of the heart, indicating sites of ganglionic stimulation in the present study. Red triangles indicate the location of neuronal clusters and epicardial ganglia. Ao = aorta; CS = coronary sinus; CV = caudal vein; DRA = dorsal right atrial subplexus; Lau = left auricle; LC = left coronary subplexus; LCV = left cranial vein; LD = left dorsal subplexus; LNC = left neuronal cluster; LPV = left pulmonary vein; LV = left ventricle; MD = middle dorsal subplexus; MPV = middle pulmonary vein; PT = pulmonary trunk; RAu = right auricle; RC = right coronary subplexus; RCV = right cranial vein; RNC = right neuronal cluster; RPV = right pulmonary vein; RV = right ventricle; VLA = ventral left atrial subplexus; VRA = ventral right atrial subplexus.

Modified from Saburkina et al.^21^

Stimulation of epicardial ganglia was applied within the 4 regions (Figure 1) using the topographical map published previously.^21^ These regions included (1) left neuronal complex (LNC), (2) right neuronal complex (RNC), (3) right atrial ganglionated plexi (RAGP), and (4) region between the middle pulmonary veins and the caudal vena cava (vena caudalis; “inferior vena cava”) (PVCV). Nicotine 0.1 mg in 10 μL saline was directly injected into loci within LNC, RNC, and PVCV and nicotine 0.1 mg in 100 μL saline^3^ into loci within RAGP to ensure a larger area of infiltration.

### Electrical stimulation of intrinsic cardiac ganglia

Electrical stimulation was applied within the 4 regions (Figure 1) using a custom-made bipolar silver electrode (0.5 mm diameter, Advent Research Materials Ltd, Oxford, UK). Electrical stimulation was delivered using a single-channel constant-voltage square-pulse stimulator (SD9, Grass Instruments, Astro-Med, Slough, UK) connected via a constant-current stimulator (DS7A, Digitimer Ltd, Welwyn Garden City, UK). Responses to stimulation were recorded at stimulation frequencies between 10 and 50 Hz (stimulus strength: 50% of the cardiac pacing threshold) by using a pulse duration of 0.1 ms.^22^

### Protocols and pharmacological agents

The effects of nicotinic and electrical stimulation were determined both during sinus rhythm and constant cardiac pacing. To determine which types of autonomic receptors were involved in the cardiac responses, protocols were repeated in the presence of pharmacological blockers (see the Supplement).

### Signal measurements and analysis

Functional responses were recorded with a PowerLab 16 channel system and digitized at 2 kHz using Chart and Scope software (ADInstruments Ltd., Chalgrove, UK) (see the Supplement).

### Immunohistochemical analysis

In addition to studying the influence of spatially divergent ganglia on cardiac electrophysiology, a further 18 animals were used for immunohistochemical analysis. Immunofluorescent labeling for choline acetyltransferase (ChAT), tyrosine hydroxylase (TH), and neuronal nitric oxide synthase (nNOS) antibodies was performed using whole mount atrial and ventricular preparations and subsequent microscopic and quantitative analysis completed (see the Supplement).

### Chemicals

Unless stated, all chemicals were purchased from Sigma Aldrich, UK.

### Statistical analysis

Data analysis was performed using GraphPad Prism7 software. Statistical comparisons were made using Student paired *t* tests or 1- or 2-way analysis of variance, where appropriate, with Bonferroni post hoc test. Data are presented as mean ± SEM. *P* < .05 was considered significant.

## Results

### Effects of nicotinic stimulation of cardiac intrinsic ganglia

Nicotine was applied in a total of 14 hearts at a number of sites within the regions shown in Figure 1: LNC in all 14 animals; RNC, RAGP, and PVCV in 13, 11, and 6 animals, respectively.

#### Chronotropic and dromotropic responses after nicotine application

**Figure 2:**
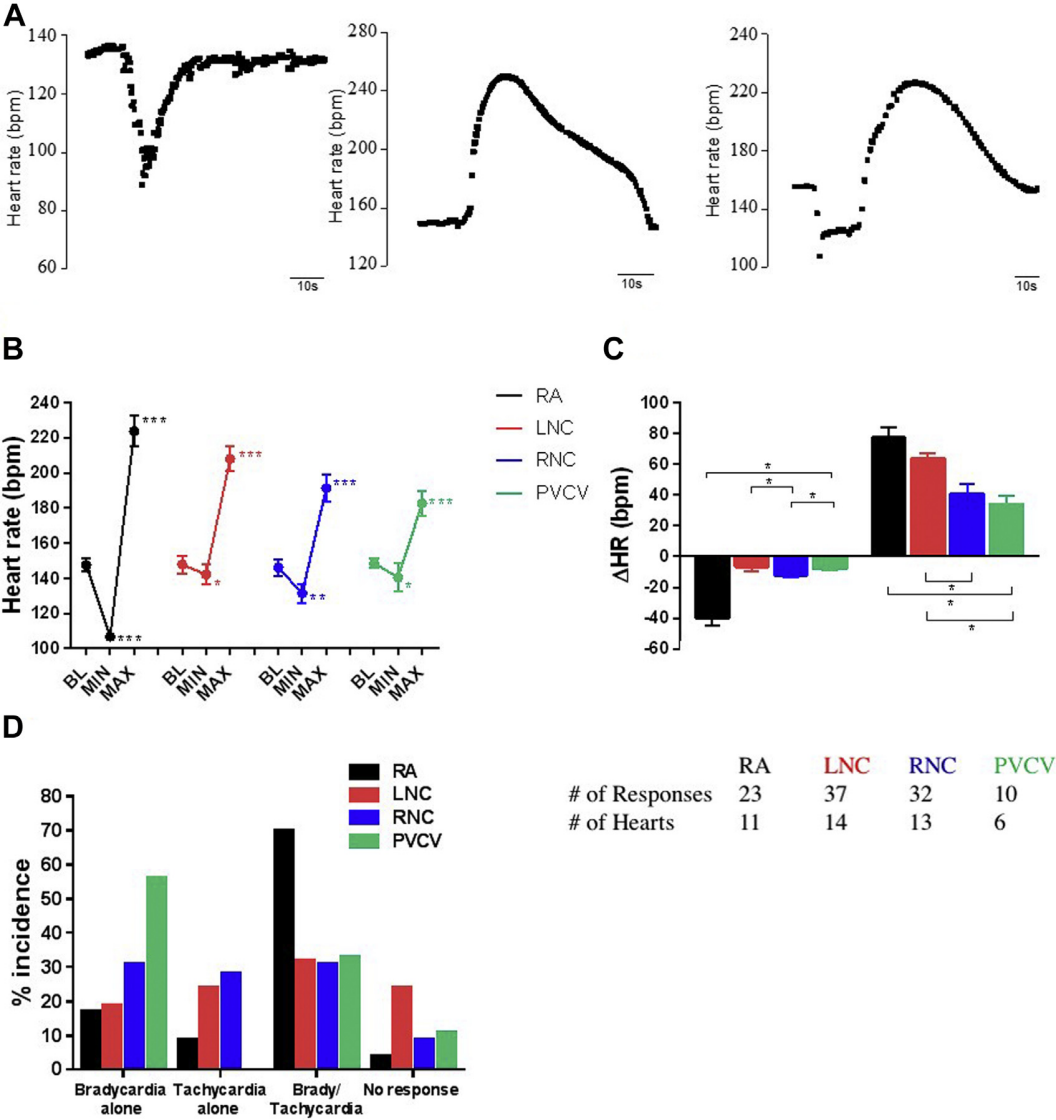
Quantification of nicotine-induced HR responses. **A:** Characteristic chronotropic responses to nicotinic application at individual cardiac regions known to contain ganglia. **B:** HR at BL and during bradycardia (MIN) and tachycardia (MAX) phases of the response. **C:** ΔHR of each phase. ^∗^*P* < .05, ^∗∗^*P* < .01, ^∗∗∗^*P* < .001 vs corresponding BL. **D:** Incidence of each HR response according to response type and region. The table given at the end presents the number of responses (nicotine applications) and hearts studied. BL = baseline; HR = heart rate; ΔHR = change in heart rate; LNC = left neuronal cluster; MAX = HR during tachycardia phases of the response; MIN = HR during bradycardia phases of the response; PVCV = pulmonary vein-caudal vein; RA = right atrial; RCV = right cranial vein.

After nicotine application, changes in heart rate (HR) occurred in 3 distinct patterns: (1) bradycardia alone, (2) tachycardia alone, and (3) a biphasic response of bradycardia followed by tachycardia (Figure 2). No region contained sites that produced a single type of HR change. At PVCV and RAGP, bradycardia and biphasic HR responses prevailed.

Nicotine applied to RAGP produced the strongest bradycardia or tachycardia, while LNC produced the smallest HR decrease and PVCV the smallest HR increase (Figure 2).

**Figure 3:**
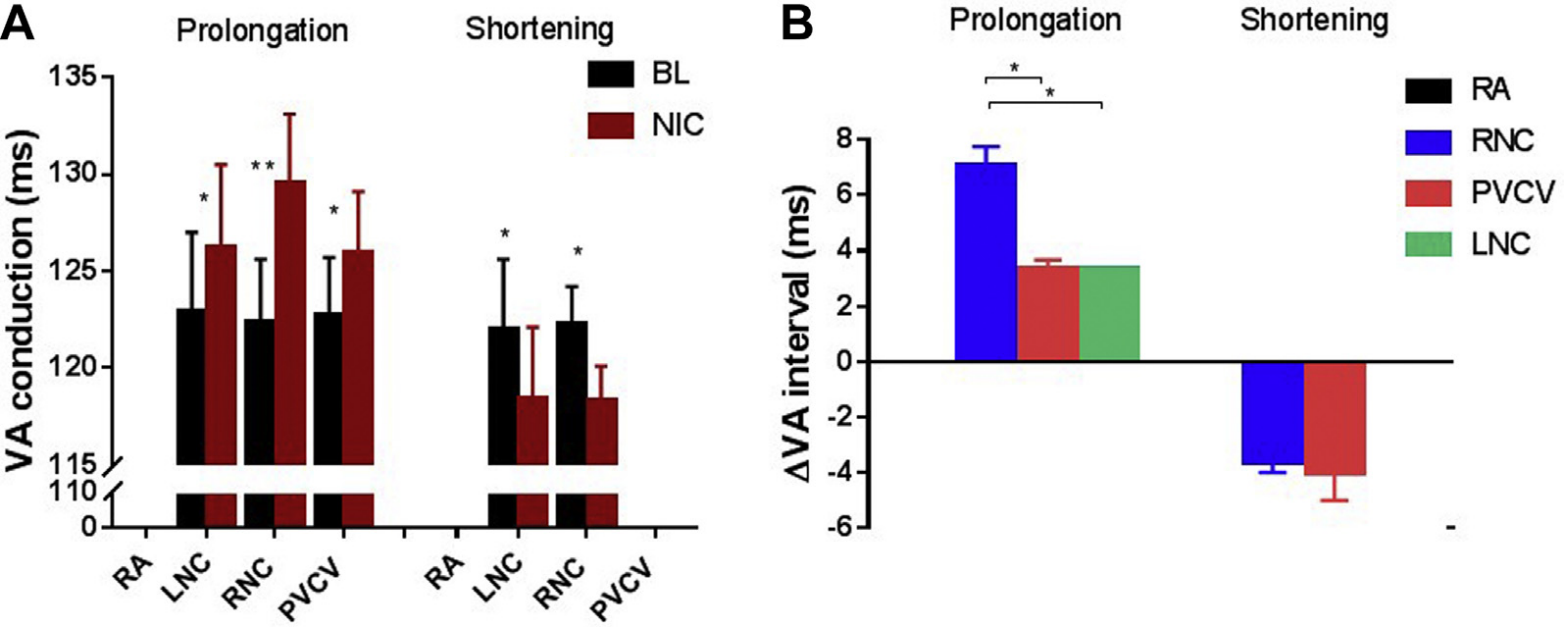
HR independent changes in atrioventricular conduction during constant right ventricular pacing. **A:** Mean data representing the interval from ventricular pacing to atrial electrogram activation at BL and after NIC application. **B:** Mean data representing the change in VA interval calculated from data in panel A. BL = baseline; HR = heart rate; LNC = left neuronal cluster; NIC = nicotine; PVCV = pulmonary vein-caudal vein; RA = right atrial; RCV = right cranial vein; VA = ventriculo-atrial; ΔVA = the change in VA from BL. ^∗^*P* < .05, ^∗∗^*P* < .01 vs BL.

During ventricular pacing, nicotine applied to RAGP sites failed to elicit any change in ventriculo-atrial (VA) conduction while nicotine applied to PVCV produced a prolongation in 60% of cases, with no effect in the remaining 40%. A combination of VA prolongation and VA shortening was observed when stimulating LNC and RNC, but there was a larger proportion resulting in VA prolongation (65% vs 35%) at RNC. Quantitatively, there was no difference in VA shortening when stimulating LNC/RNC. There was however a greater degree of VA prolongation at RNC compared to others, with LNC and PVCV sites being equipotent (Figure 3).

### Effects of electrical stimulation at sites in the cardiac intrinsic ganglionated plexus

Electrical stimulation of intrinsic cardiac ganglia was examined in 14 animals: RNC in all 14 animals; LNC, RAGP, and PVCV in 8, 9, and 8 animals, respectively.

#### Chronotropic responses to electrical stimulation

**Figure 4:**
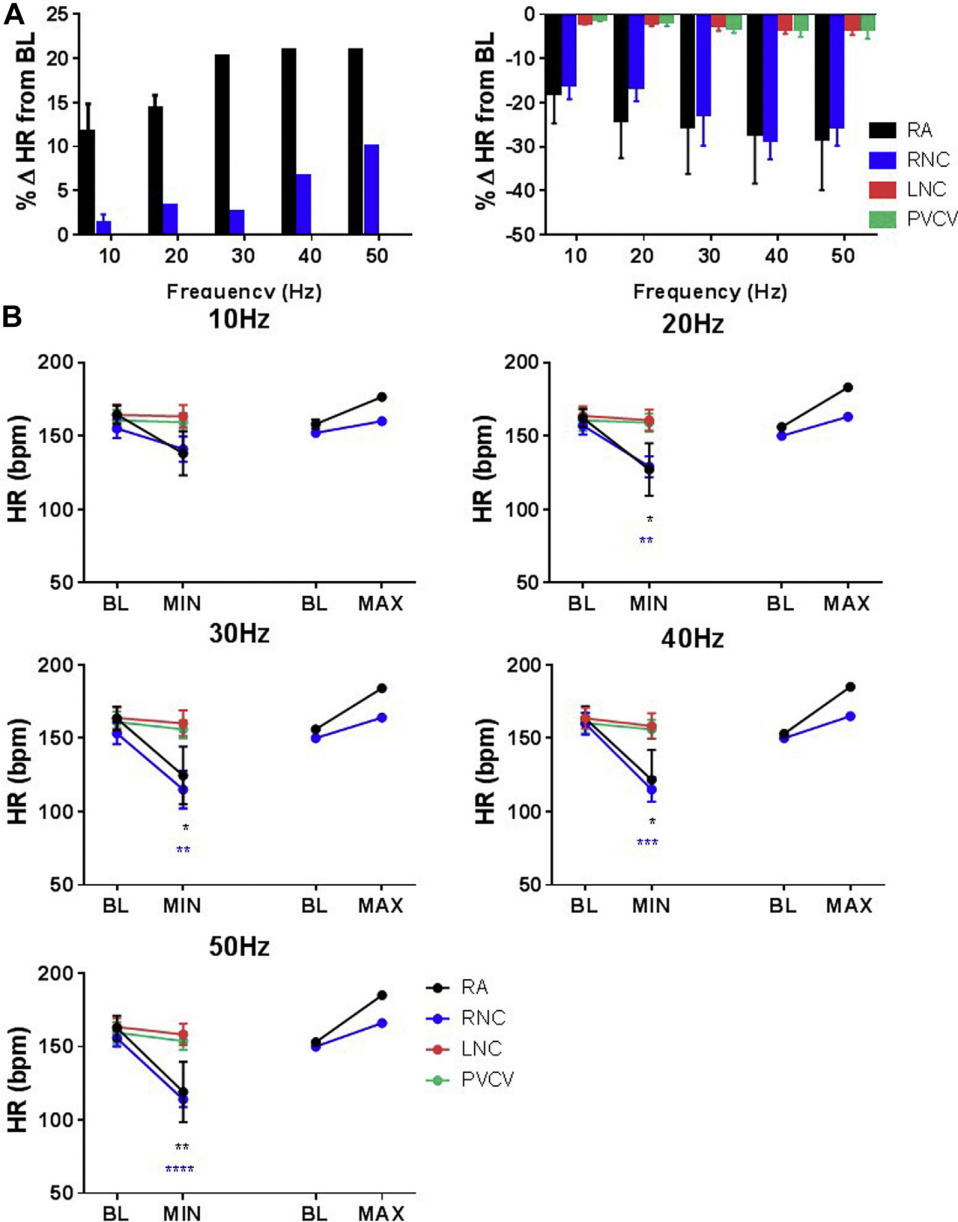
HR responses induced by electrical stimulation at sites in RA, RNC, LNC, and PVCV. **A:** Comparison of %ΔHR during electrical stimulation at different regions. **B:** Comparison of maximum reductions and maximum increases in HR compared to BL during electrical stimulation at different frequencies—10, 20, 30, 40, and 50 Hz—at sites in different regions—RA (*black*), RNC (*blue*), LNC (*red*), and PVCV (*green*). Data are presented as mean ± SEM. BL = baseline; HR = heart rate; %ΔHR = percent change in heart rate; LNC = left neuronal cluster; MAX = maximum increase in HR compared to BL; MIN = maximum reduction in HR compared to BL; PVCV = pulmonary vein-caudal vein; RA = right atrial; RCV = right cranial vein. ^∗^*P* < .05, ^∗∗^*P* < .01, ^∗∗∗^*P* < .001,^∗∗∗∗^*P* < .0001 vs BL HR.

The effects of electrical stimulation (10–50 Hz) were examined at 56 sites. Decreases in HR occurred on stimulation at sites in all regions tested. As with nicotine, electrical stimulation at sites in ganglia could elicit 3 types of response. Tachycardia occurred at 5% of sites tested electrically (3 of 14), all at RA. The most pronounced HR decreases were produced at RNC and RA regions (−25.5% ± 4.2% and −21.0% ± 12.0%, respectively) (Figure 4). A single biphasic response was noted at 1 locus within RNC (155 to 121 to 220 beats/min).

At frequencies ≥20 Hz, there was a significant reduction in the mean HR during stimulation within RNC and RA (Figure 4). Small HR reductions were noted during stimulation within LNC and PVCV (Figure 4).

#### Left ventricular pressure and monophasic action potential duration changes

There was no statistically significant difference in mean left ventricular pressure during electrical stimulation compared to baseline between sites stimulated in each of the 4 regions as well as no significant difference in percent change in left ventricular pressure when each group was compared. There was also no significant change in monophasic action potential duration.

#### Dromotropic changes during electrical stimulation

**Figure 5:**
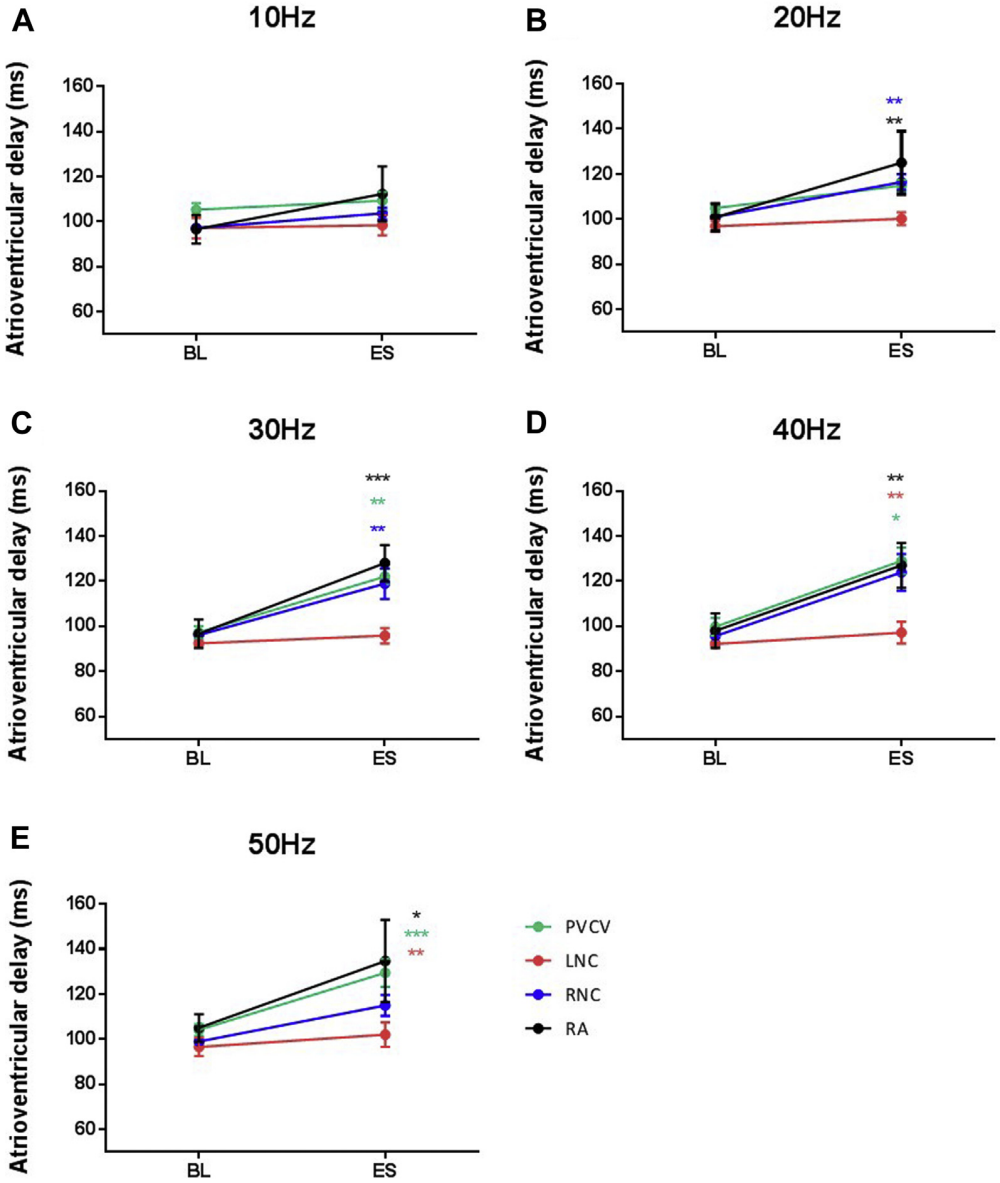
ES–induced effects on atrioventricular conduction. Mean data representing the average change in atrioventricular delay during electrical stimulation at sites in intrinsic cardiac ganglia—PVCV (n = 9), LNC (n = 7), RNC (n = 27), and RA (n = 4)—at different frequencies—(**A**) 10 Hz, (**B**) 20 Hz, (**C**) 30 Hz, (**D**) 40 Hz, and (**E**) 50 Hz. Data are presented as mean ± SEM. BL = baseline; ES = electrical stimulation; LNC = left neuronal cluster; PVCV = pulmonary vein-caudal vein; RA = right atrial; RCV = right cranial vein. ^∗^*P* < .05, ^∗∗^*P* < .01, ^∗∗∗^*P* < .001.

Atrioventricular conduction (AVC) during constant atrial pacing was measured from the pacing stimulus to the beginning of ventricular monophasic action potential (MAP). At 9 sites out of 54 tested, significant prolongations of AVC ranging from >20 ms to atrioventricular block were measured. Significant atrioventricular prolongation occurred during electrical stimulation of at least 1 locus within RA, RNC, and PVCV. Stimulation within LNC failed to elicit changes in AVC. The largest changes in AVC occurred during stimulation within RA, the only region where electrical stimulation elicited atrioventricular block (Figure 5).

### Pharmacology of chronotropic responses to nicotinic and electrical stimulation

**Figure 6:**
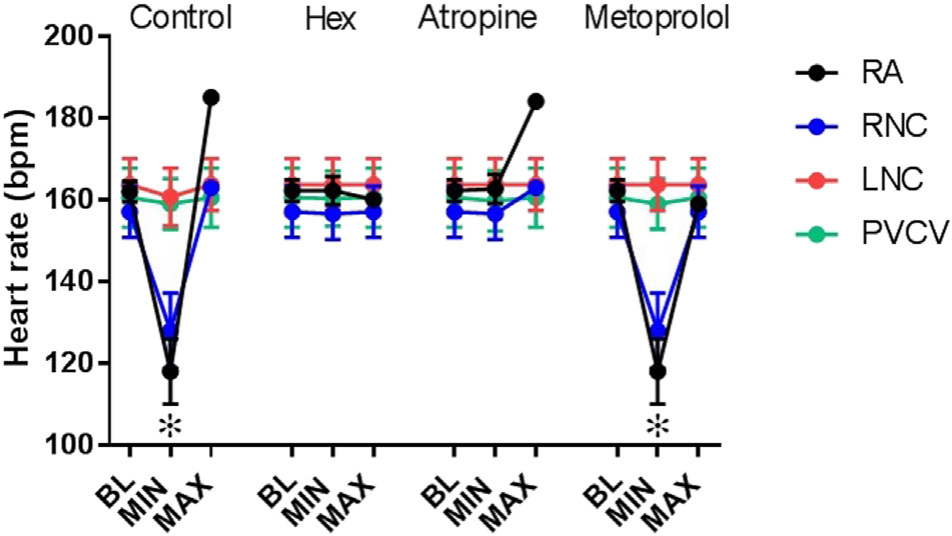
Pharmacological autonomic blockade of HR responses to electrical stimulation. HR response during control and in the presence of atropine (0.1 μM^15^), metoprolol (1.8 μM^15^), and hexamethonium (0.5 mM^23^). Electrical stimulation applied at either LNC, PVCV, RA, or RNC. Data are presented as mean ± SEM. BL = baseline; HR = heart rate; LNC = left neuronal cluster; MAX = maximum increase in HR compared to BL; MIN = maximum reduction in HR compared to BL; PVCV = pulmonary vein-caudal vein; RA = right atrial; RCV = right cranial vein; RNC = right neuronal cluster. ^∗^*P* < .001 vs BL HR.

The effects of pharmacological blockade are presented in Figure 6. All HR responses to electrical or nicotinic stimulation were abolished in the presence of hexamethonium. The bradycardic responses were blocked by atropine, and the tachycardic responses were blocked by metoprolol. None of these pharmacological antagonists had effects on baseline values during electrical stimulation, especially evidenced by lack of changes during tests at LNC and PVCV (Figure 6).

#### Immunohistochemical characterization and distribution of the rabbit ICNS

The location of intrinsic cardiac neuronal somata and ganglia positive for ChAT, TH, and/or nNOS was reproducible from animal to animal in both atrial and ventricular preparations. Despite this, the precise anatomical location and size of individual ganglia varied between hearts. Neurons immunoreactive (IR) only for ChAT, TH, or nNOS were consistently located at the heart hilum, roots of the pulmonary veins, and root of the right cranial vein (RCV), corresponding to neurons previously identified in these regions.^21^

**Figure 7:**
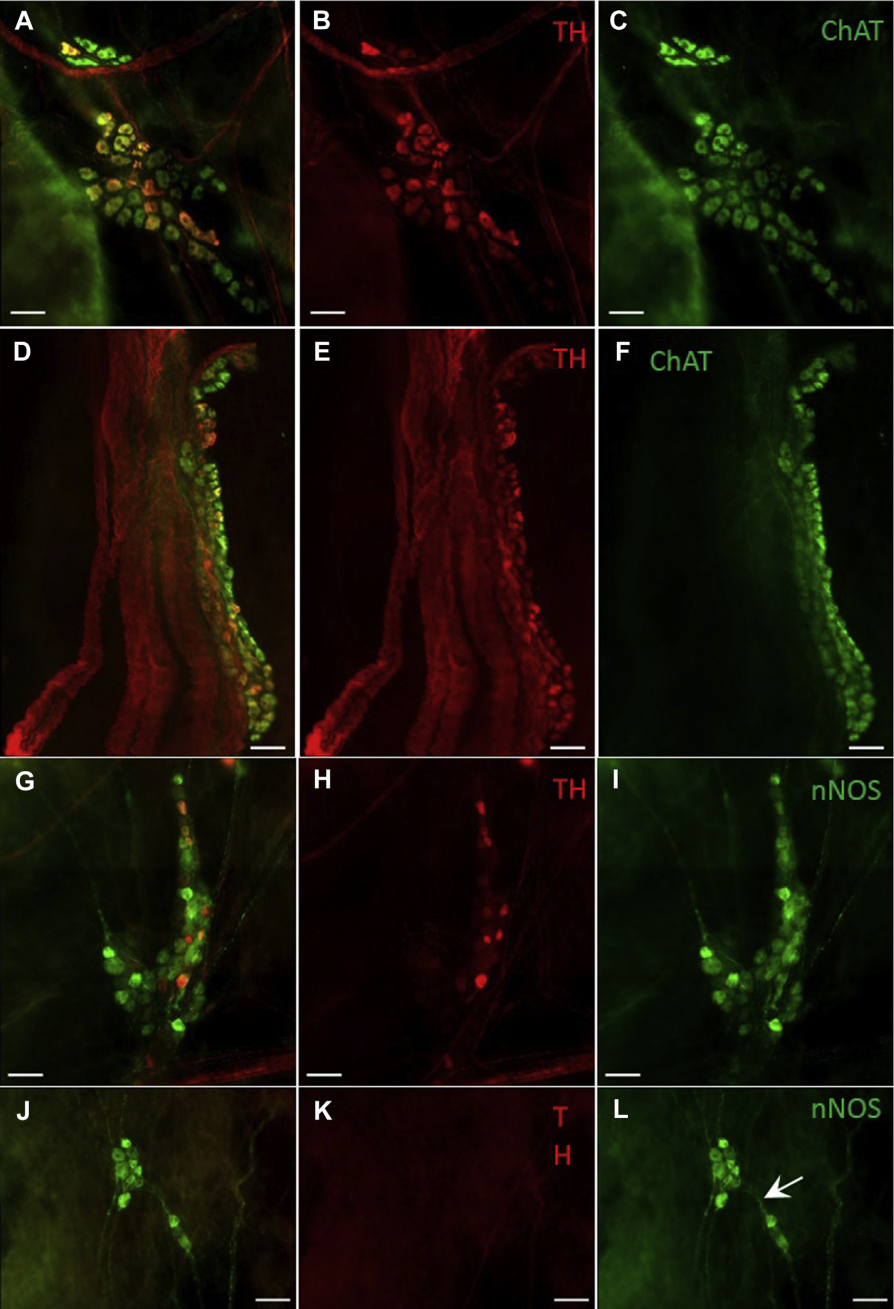
Microphotographs illustrating the predominance of cholinergic (ChAT-IR) neurons within ganglia. **A–C:** Microphotographs of ganglia containing both ChAT-IR and TH-IR neurons located within the RNC of the rabbit ICNS. **D–F:** Microphotographs illustrating nerves accessing the heart on the medial side of the root of RCV, where TH-IR nerve fibers predominate. **G–I:** Microphotographs illustrating the presence of both TH-IR and nNOS-IR neurons at the root of RCV. **J–L:** Illustration of a smaller ganglion located in close proximity to ganglia shown in images G and H and containing only nNOS-positive neurons. Note the thin nNOS-IR nerve fibers (*white arrow*) connecting 2 small neighboring ganglia. Scale bars represent 100 μm. ChAT = choline acetyltransferase; IR = immunoreactive; nNOS = neuronal nitric oxide synthase; RCV = right cranial vein; RNC = right neuronal cluster; TH = tyrosine hydroxylase.

ChAT-IR neurons were significantly more abundant than all other phenotypes studied (1946 ± 668 neuronal somata per heart) (Table 1). ChAT-IR somata formed large ganglia, with a large proportion of neuronal somata of LNC and RNC being cholinergic. In comparison, markedly smaller numbers of neurons IR for TH or nNOS were identified (326 ± 106 and 111 ± 20, respectively) (Table 1). Neurons IR for TH or nNOS were often found dispersed throughout larger ganglia containing numerous cell phenotypes (Figure 7). Neurons that were only nNOS positive were also identified forming smaller ganglia (Figure 7L), both atrially and on the ventricular epicardium. In addition to singularly labeled cells (ie, ChAT, TH, *or* nNOS only), numerous ganglia containing biphenotypic neurons were observed (Figure 7), which were consistent and dispersed throughout ganglia.

**Table 1:** Mean number, range, and size of immunohistochemically distinct intrinsic cardiac neuronal somata identified in whole mount preparations of the rabbit

Variable	ChAT (n = 9)	TH (n = 10)	nNOS (n = 9)	ChAT/TH (n = 4)	ChAT/nNOS (n = 3)	TH/nNOS (n = 5)
Number of somata per heart, mean	1946 ± 668	326 ± 106	111 ± 20	616 ± 161	203 ± 58	112 ± 36
Number of somata per heart, range	1014–3240	35–854	54–193	340–899	107–308	22–211
Area of neurons (μm^2^)	557 ± 31	507 ± 35	478 ± 32	515 ± 30	600 ± 48	519 ± 45
Short axis (μm)	25.0 ± 0.9	22.6 ± 1.1	23.1 ± 1.3	23.2 ± 0.4	25.9 ± 1.7	22.6 ± 1.1
Long axis (μm)	32.2 ± 1.4	29.4 ± 1.6	29.6 ± 1.7	31.0 ± 1.0	32.2 ± 2.7	29.6 ± 1.6

ChAT = choline acetyltransferase; nNOS = neuronal nitric oxide synthase; TH = tyrosine hydroxylase.

In all hearts examined, intrinsic cardiac ganglia were regularly interconnected by thinner commissural nerves reactive for ChAT, TH, and nNOS. Numerous thin commissural nerves were recognized epicardially connecting ganglia on the heart hilum, at roots of the pulmonary veins, and at the root of RCV, extending between individual ganglia (Figure 7).

Larger ChAT-IR and TH-IR nerve fibers passed epicardially over the venous portion of the heart hilum, extending to numerous ganglia. In all preparations examined for ChAT and TH immunoreactivity, larger bundles of nerve fibers were present with ChAT-IR axons extending in parallel to TH-IR axons. The majority of large neural bundles, however, contained primarily TH-IR axons including nerves accessing the heart on the medial side of the root of RCV (Figures 7D–7F). These neural bundles ran adjacent to large ganglia where the predominant neurochemical phenotype was cholinergic (Figure 7).

## Discussion

This is the first study to investigate the functional effects of activating sites within ganglia of the ICNS in rabbit hearts. Furthermore, unlike previous studies on dogs,^24^ tests were performed using a Langendorff-perfused heart preparation to avoid the influence of extrinsic autonomic nerves and circulatory factors. The results of this study also characterize the immunohistochemical profile of the rabbit atrial epicardial network as a whole for the first time.

Using pharmacological and electrical stimuli applied to discrete sites, the results indicate that these stimuli activated neurons and synapses that lie in the intrinsic neural plexus located within RA, RNC, LNC, and PVCV (Figure 1). These are regions within which stimulation has previously been shown to modify sinoatrial rate and AVC in anesthetized dogs^2^ or mice.^22^ The similarity of the present data from the isolated perfused rabbit heart would appear to strengthen the conclusions of the latter studies: that the ICNS can independently influence cardiac functions. However, the electric current will activate afferent and efferent axons traversing ganglia as well as nearby neuronal somata and dendrites. Therefore, in order to discriminate the elements affected at different sites, we separately tested the action of nicotine, a postsynaptic receptor stimulant. At several loci within both RNC and LNC (Figures 2 and 3), both types of stimuli could evoke similar cardiac effects such as HR increase alone, HR decrease alone, or a biphasic effect as well as effects on AVC. Therefore, it appears most likely that the effects of either stimulus on cardiac activity were caused by activating ICNS neurons acting as components of the efferent limb of the ICNS rather than extrinsic nerve terminals. Despite similar cardiac responses being elicited between individual hearts, known anatomical variability of the ganglionated nerve plexus^21^ as well as the dispersed distribution of intrinsic ganglionic cells between different ganglia and neuronal clusters is hypothesized to be the reason for the highly variable nature of responses to stimulation.^3^

By performing this study using the Langendorff-perfused heart preparation, it is possible to interpret the functional effects of electrical and nicotinic stimulation in the absence of extrinsic autonomic inputs. The application of nicotine at discrete sites produced responses in line with those shown previously in anesthetized canines.^3^ Previous functional studies investigating the effects of nicotinic stimulation of intrinsic cardiac ganglia illustrated similar responses induced in both in situ intact preparations and acutely decentralized preparations.^3^, ^25^ By comparison, a previous study using the autotransplant model to investigate the effect of loss of the intrinsic-extrinsic cardiac autonomic interaction indicated that neurons found in acutely transplanted heart preparations were still capable of considerable cardiac augmentation, albeit with slightly reduced responses compared with those noted before transplantation,^26^ further reiterating the involvement of intrinsic cardiac ganglia in the contribution to modulation of cardiac function, independent of central neural inputs.

Electrical and nicotinic stimulation of ganglia within the RA region demonstrated their dominance compared to left-sided ganglia in HR control. This is consistent with previous functional studies in dogs^3^ and congruent with anatomical data from several species and with the present study in rabbits. This accords with anatomical studies in rabbits,^21^ which reported RA ganglia located along nerves extending epicardially from the right neuronal cluster, along with minute epicardial ganglia coursing toward the region of the sinus node. In comparison, stimulation of ganglia at the limits of the heart hilum had little or no effect on hemodynamic parameters or monophasic action potential duration. It has previously been comprehensively demonstrated that myocardial innervation of the rabbit ventricles occurs mainly via the epicardial nerve plexus (ENP)^27^; however, the results shown here draw attention to the likely possibility that the effects of the ENP on the ventricular myocardium are highly localized and further investigation into the complete involvement of the ENP in ventricular electrophysiology is warranted.

Notable differences between electrical and nicotinic stimulation were evident in this study. The effects of the electric current on the left neuronal cluster were absent or weak. The reason for this is unclear, but may have been a consequence of the short pulse duration of 0.1 ms and could be understood to suggest that either the current strength was insufficient or there were few neuronal somata in these loci. Unfortunately, a direct comparison between both methods of stimulation at the same site could not be made because of technical difficulties in placing electrodes or micropipettes at the same locations. However, the results serve to emphasize the complexity of the ICNS and the individual capabilities of specific ganglia.

The incidence of biphasic effects is unsurprising since stimuli were applied to ganglia in which networks of connected neurons are present as can be seen in whole mount preparations. Spread of the chemical or electric current from a micropipette or a bipolar electrode would be difficult to restrict in such an environment. Nonetheless, the fact that on many occasions bradycardia alone or tachycardia alone was observed suggests that neurons at these loci were more isolated or in smaller functionally similar groups, which concurs with the neuroanatomy.

An interesting observation was the significant atrioventricular prolongation evoked at PVCV (commonly around the root of the caudal vein or inferior vena cava). This was a region from which small HR changes could be elicited, which suggests that these ganglia play a dominant role in atrioventricular nodal innervation rather than sinoatrial nodal control, correlating with previous findings in dogs.^3^, ^28^ Taken together, these data support the notion that the mammalian heart has its own nervous system, whereby groups of neurons connect with spatially diverse intrinsic cardiac ganglia to influence myocardial activity and function.^6^, ^29^

The present study also tested the synaptic interaction and junctional receptor transmission involved in the cardiac responses. We showed that the effects of the chemical or electric current observed depended on cholinergic nicotinic ganglion transmission and that bradycardiac effects were mediated by cholinergic muscarinic receptors while tachycardic effects were mediated via postganglionic β-adrenoreceptor activation. These observations are unsurprising but are the first of their kind. Unlike in previous studies where the focus has been on either the quantitative or morphometric characterization^21^ of neuronal somata related to distinct regions of the heart,^12^, ^30^ the present study investigates and characterizes the atrial epicardial network in the rabbit as a whole. The immunohistochemical study performed using whole mount preparations of atria and ventricles demonstrated a wide phenotypic complexity of the intrinsic cardiac nerve plexus, indicating that the cholinergic and adrenergic connections provide only a brief glimpse of the functional neurochemistry of the ICNS.^8^, ^9^, ^11^, ^12^, ^31^, ^32^ In this context, of particular significance was the clear demonstration of neurons IR only for nNOS, with nerve fibers connecting with neurons in linking ganglia (Figure 7L). Exploration of the physiology of these nNOS neurons requires further study, but there is an important correlation of the present data to our earlier studies performed using isolated innervated rabbit heart preparations, showing the protective effects of cervical vagus nerve stimulation against ventricular fibrillation to be dependent only on nitrergic postganglionic fibers.^23^, ^33^, ^34^ The results shown here are the first to provide anatomical support for the likely involvement of these neurons in the neurocardiac effects.

## Conclusion

Over recent decades it has become increasingly evident that the ICNS is a key network involved in the cardiac neuronal hierarchy. The results of this study reveal the previously uncharacterized neurochemical phenotype of the atrial epicardial network as well as draw attention to a significant capability of clusters of neurons in independently and selectively modulating cardiac electrophysiology. This study therefore provides an important basis for further exploration into the involvement and potential therapeutic target of the ICNS in cardiac disease.
