# Supplementary material for: Electrophysiological effects of nicotinic and electrical stimulation of intrinsic cardiac ganglia in the absence of extrinsic autonomic nerves in the rabbit heart
Source: Heart Rhythm. 2018 Nov;15(11):1698–707. doi: 10.1016/j.hrthm.2018.05.018 (PMC6207532; doi:10.1016/j.hrthm.2018.05.018)
Supplement: Supplement [file mmc1.docx]

**Supplementary Material**

**The electrophysiological effects of nicotinic and electrical stimulation of intrinsic cardiac ganglia in the absence of extrinsic autonomic nerves in the rabbit heart**

Allen E, Coote JH, Grubb BD, Batten TFC, Pauza DH, Ng GA, Brack KE

**Animal Preparation**

### All animals were pre-medicated with ketamine (Ketaset,10 mg/kg,Fort Dodge,UK), medetomidine hydrochloride (Sedator,0.2 mg/kg,Dechra,UK) and butorphanol (Torbugesic,0.05 mg/kg s.c.,Fort Dodge,UK). Following stable sedation, animals were sacrificed with an overdose of pentobarbitone sodium (Sagatal,Rhone Merieux,UK;111mg/kg body weight, i.v.) containing heparin (1000 IU,Multiparin,UK) delivered via the marginal ear vein.

### Isolation of the non-innervated heart preparation

### Langendorff perfusion

Hearts were perfused with Tyrode solution containing Na^+^138.0; K^+^4.0; Ca^2+^1.8; Mg^2+^1.0; HCO_3_^-^24.0; H_2_PO_4_^-^0.4; Cl^-^124.0; Glucose11.0(mM). The solution was maintained at 37°C (pH7.4) by continuous bubbling with Carbogen [95%O_2_/5%CO_2_]. A 1mm ID, 2mm OD polypropylene catheter (Porlex,Kent,UK) was inserted through the left ventricular (LV) apex for Thebesian venous effluent drainage. Hearts were instrumented to record left ventricular pressure(LVP), coronary perfusion pressure(PP) and ventricular monophasic action potentials(MAP) at the apex and base with MAP contact electrodes (73-0150,Harvard Apparatus,Kent,UK) using a custom made DC-coupled high input impedance differential amplifier (Joint Biomedical Workshop,University of Leicester,UK).^[16]^

**Protocols**

The effects of nicotine and electrical stimulation were determined both during sinus rhythm or constant cardiac pacing. In order to account for the maximum anticipated increase in heart rate (HR) based on observations during preliminary investigations, hearts were paced at a 240ms cycle length at double the pacing threshold.

**Pharmacological Agents**

To determine which types of autonomic receptors were involved in the cardiac responses, protocols were repeated in the presence of the muscarinic (M2) receptor antagonist, atropine (0.1μM^[16]^), the β-adrenergic receptor blocker, metoprolol (1.8μM^[16]^) and the autonomic ganglia blocker, hexamethonium (0.5mM^[23,24]^). Each agent was dissolved in a small volume of Tyrode solution before being added to the perfusate, with measurements being made 5-10 minutes following exposure and following a period of washout (to confirm effects were due to application of a pharmacological agent).

### Signal measurements and analysis

Functional responses were recorded with a PowerLab 16 channel system and digitised at 2kHz using Chart and Scope software (ADInstruments Ltd). During sinus rhythm, HR and LV pressure were measured along with contact MAP duration measured at 90% repolarisation (MAPD_90_); calculated from an average of 10 cardiac cycles during baseline and subsequently during the steady state response. The PR Interval was calculated using AD instruments ECG measuring software. During constant atrial pacing, parameters were measured from averaged data taken from 30 cardiac cycles during steady state using AD instruments Scope software. The time from the cardiac pacing stimuli to the activation of atrial / ventricular electrical signal was used to determine AV delay. MAPD_90_ was measured using custom-made NewMap analysis software (Dr F Burton, University of Glasgow).^[16]^

**Immunohistochemical analysis**

**Whole-mount preparations**

Following euthanasia, the chest was opened by bilateral thoracotomy to expose the lungs and heart and the pericardium was cut to allow access to the heart. The caudal vein (vena caudalis; ‘inferior vena cava’) was identified and cannulated and a cannula inserted via an incision at the superior edge of the left atrium, thereby perfusing the right and left sides of the heart respectively. Hearts were perfused with ice-cold phosphate-buffered saline (PBS; 0.01M, 4˚C, pH7.4) and pressure-inflated *in situ* ^[1]^. Hearts were removed from the chest, placed into ice-cold PBS (pH 7.4, 4^o^C) and the surrounding pericardium, mediastinal fat were removed and pulmonary vessels sectioned. Hearts were prefixed using 4% PFA (0.01M PB, pH7.4, 30 minutes), followed by incubation in a hyaluronidase solution (PBS, 0.5mg/100ml, 60 minutes).

**Immunolabelling procedure**

The walls of the atria and interatrial septum were separated from the ventricles along the atrioventricular groove and pinned flat. In order to assess the distribution of ventricular neurons, the wall of the conus arteriosus and left ventricle was dissected and pinned, allowing for subsequent washes (3 x 10minutes) in ice cold PB (0.01M, pH7.4, 4^o^C). Whole mount preparations were incubated in a blocking buffer (0.01M PB containing 10% normal horse serum (Vector Laboratories, UK) and 0.5% Triton X-100 (Sigma Aldrich, UK)) for 30 minutes. Preparations were then incubated in a double primary antibody mixture (Table. 1) (antibodies were diluted in a solution of 0.01M PB containing 10% normal horse serum and 0.5% Triton X-100) for 48 hours at 4˚C in a humidity chamber ^[25]^.

After a washing step (3 x 10 minute washes in 0.01M PB), all sections were incubated in the combination of corresponding secondary antibodies (supplementary table.1) for 2 hours (room temperature). After a final wash step (3 x 10 minute washes in 0.01M PB) each section was mounted using Vectashield Hardset Mounting Medium (Vector Laboratories, UK), covered with a coverslip and sealed using clear nail varnish ready for microscopic analysis.

**Microscopic examination and quantitative analysis**

Immunohistochemically stained neural structures were visualised and imaged using an upright fluorescence microscope (Axioscope Imager.Z2, Carl Zeiss Ltd, UK). Images were captured using a digital camera (AxioCam HRc, Carl Zeiss Ltd, Cambridge, UK). Stereoscopic examination was achieved between x10-40 magnification. Overall topography was constructed offline and image examination and analysis was completed using ZEN software (Carl Zeiss Ltd, Cambridge, UK) and Image J 1.49v software. For counting and identifying, neuronal cell bodies and their areas were systematically scanned over the whole mount.

**Supplementary Table 1**

| **Primary Antibody** | **Host Species** | **Dilution** | **Catalogue Number and Supplier** |
| --- | --- | --- | --- |
| ChAT | Goat | 1:100 | AB144P Merck Millipore |
| TH | Mouse | 1:2000 | 22941 Immunostar |
| nNOS | Mouse | 1:300 | SC-5302 Santa Cruz |
| nNOS | Sheep | 1:1000 | Gift from Professor P. Emson, University of Cambridge |
| **Secondary Antibody** |  |  |  |
| Anti-goat (AF594) | Donkey | 1:300 | Jackson Immunoresearch |
| Anti-goat (FITC) | Donkey | 1:100 | Jackson Immunoresearch |
| Anti-mouse (AF594) | Donkey | 1:300 | Jackson Immunoresearch |
| Anti-mouse (FITC) | Donkey | 1:100 | Jackson Immunoresearch |

**Table 1.** Primary and secondary antisera used within this study.
